# (*E*)-*N*′-(5-Bromo-2-hy­droxy­benzyl­idene)-2-meth­oxy­benzohydrazide

**DOI:** 10.1107/S1600536810022002

**Published:** 2010-06-16

**Authors:** Shi-Yong Liu, Zhonglu You

**Affiliations:** a College of Chemistry & Pharmacy, Taizhou University, Taizhou Zhejiang 317000, People’s Republic of China; b Department of Chemistry, Liaoning Normal University, Dalian 116029, People’s Republic of China

## Abstract

In the title compound, C_15_H_13_BrN_2_O_3_, the mol­ecule adopts an *E* configuration about the C=N bond and the two benzene rings form a dihedral angle of 20.3 (3)°. In the mol­ecule, there are two intra­molecular hydrogen bonds, *viz*. O—H⋯N and N—H⋯O, involving the hy­droxy substituent, the meth­oxy O atom and the hydrazide NH group and N atom. In the crystal structure, mol­ecules are linked through N—H⋯O hydrogen bonds, forming chains propagating along [010].

## Related literature

For background to hydrazones and their medicinal applications, see: Hillmer *et al.* (2010 ▶); Zhu *et al.* (2009 ▶); Jimenez-Pulido *et al.* (2008 ▶); Raj *et al.* (2007 ▶); Zhong *et al.* (2007 ▶). For the crystal structures of hydrazones, see: Khaledi *et al.* (2009 ▶); Warad *et al.* (2009 ▶); Back *et al.* (2009 ▶); Vijayakumar *et al.* (2009 ▶). For similar compounds, see: Cao (2009 ▶); Xu *et al.* (2009 ▶); Shafiq *et al.* (2009 ▶).
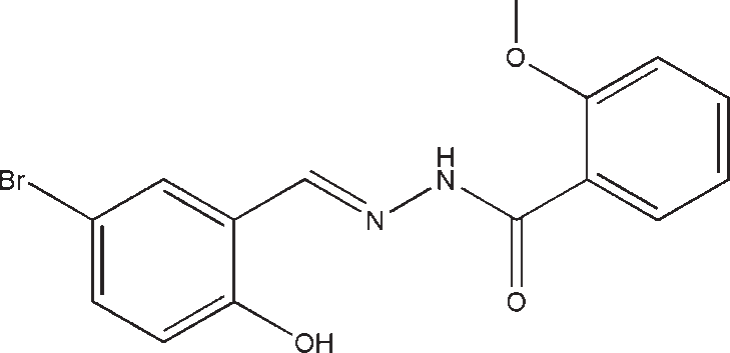


## Experimental

### 

#### Crystal data

C_15_H_13_BrN_2_O_3_
*M* *_r_* = 349.18
Orthorhombic, 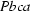

*a* = 15.587 (3) Å
*b* = 9.1281 (19) Å
*c* = 20.399 (4) Å
*V* = 2902.3 (10) Å^3^
*Z* = 8
Mo *K*α radiation
μ = 2.84 mm^−1^
*T* = 298 K
0.23 × 0.20 × 0.20 mm

#### Data collection

Bruker SMART CCD area-detector diffractometer
Absorption correction: multi-scan (*SADABS*; Bruker, 2001 ▶) *T*_min_ = 0.561, *T*_max_ = 0.600
16311 measured reflections
3154 independent reflections
1496 reflections with *I* > 2σ(*I*)
*R*_int_ = 0.074

#### Refinement

*R*[*F* ^2^ > 2σ(*F* ^2^)] = 0.043
*wR*(*F* ^2^) = 0.127
*S* = 1.00
3154 reflections
195 parameters
1 restraint
H atoms treated by a mixture of independent and constrained refinement
Δρ_max_ = 0.64 e Å^−3^
Δρ_min_ = −0.78 e Å^−3^

### 

Data collection: *SMART* (Bruker, 2007 ▶); cell refinement: *SAINT* (Bruker, 2007 ▶); data reduction: *SAINT*; program(s) used to solve structure: *SHELXS97* (Sheldrick, 2008 ▶); program(s) used to refine structure: *SHELXL97* (Sheldrick, 2008 ▶); molecular graphics: *SHELXTL* (Sheldrick, 2008 ▶); software used to prepare material for publication: *SHELXTL*.

## Supplementary Material

Crystal structure: contains datablocks global, I. DOI: 10.1107/S1600536810022002/su2186sup1.cif

Structure factors: contains datablocks I. DOI: 10.1107/S1600536810022002/su2186Isup2.hkl

Additional supplementary materials:  crystallographic information; 3D view; checkCIF report

## Figures and Tables

**Table 1:** Hydrogen-bond geometry (Å, °)

*D*—H⋯*A*	*D*—H	H⋯*A*	*D*⋯*A*	*D*—H⋯*A*
N2—H2⋯O2^i^	0.89 (1)	2.14 (2)	2.978 (4)	157 (4)
N2—H2⋯O3	0.89 (1)	2.28 (4)	2.726 (4)	111 (3)
O1—H1⋯N1	0.82	1.93	2.646 (4)	146

Symmetry code: (i) 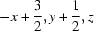.

**Fig. 1.**
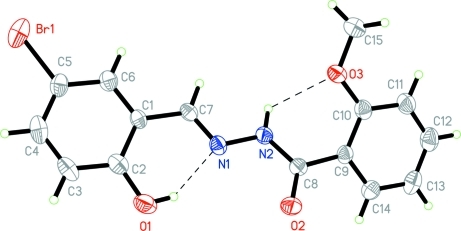
The molecular structure of the title compound, with displacement ellipsoids drawn at the 30% probability level. Hydrogen atoms are shown as spheres of arbitrary radius. Hydrogen bonds are shown as dashed lines (see Table 1 for details).

**Fig. 2.**
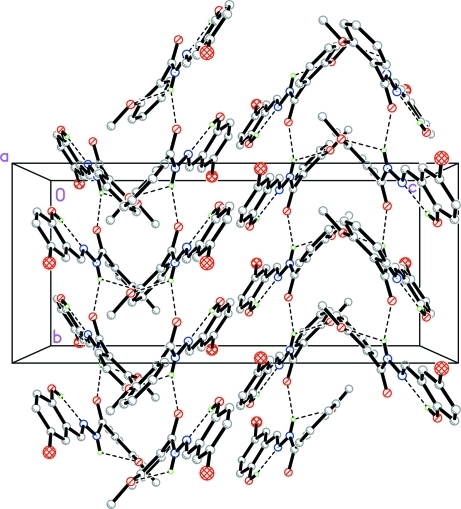
The crystal packing of the title compound, viewed along the a axis. Hydrogen bonds are shown as dashed lines (see Table 1 for details). Hydrogen atoms not involved in hydrogen bonding have been omitted for clarity.

## supplementary crystallographic information

### Comment

Considerable attention has been focused on hydrazones and their medicinal applications (Hillmer *et al.*, 2010; Zhu *et al.*, 2009; Jimenez-Pulido *et al.*, 2008; Raj *et al.*, 2007; Zhong *et al.*, 2007). The study of the crystal structures of such compounds is of particular interest (Khaledi *et al.*, 2009; Warad *et al.*, 2009; Back *et al.*, 2009; Vijayakumar *et al.*, 2009), and herein we report on the crystal structure of the new title hydrazone.

In the title molecule, illustrated in Fig. 1, the dihedral angle between the two benzene rings is 20.3 (3)°, indicating that the molecule is somewhat twisted. Atom C15 deviates from the plane of the benzene ring (C9-C14) by 0.075 (2) Å. All the bond lengths are comparable to those in similar compounds (Cao, 2009; Xu *et al.*, 2009; Shafiq *et al.*, 2009). In the molecule there are two intramolecular hydrogen bonds; O-H···N involving the hydroxyl group and the adjacent N hydrazide atom, and N-H···O involving the NH group and the adjacent O-atom of the methyl group (Table 1). The molecule has the E configuration about the C═N bond.

In the crystal structure, molecules are linked through N—H···O hydrogen bonds, to form chains running parallel to the *b* axis (Fig. 2, and Table 1).

### Experimental

The title compound was prepared by the condensation reaction of 5-bromosalicylaldehyde (0.05 mol, 10 g) and 2-methoxybenzohydrazide (0.05 mol, 8.3 g) in anhydrous methanol (200 mL) at RT. Colourless block-shaped single crystals, suitable for X-ray structure analysis, were obtained by slow evaporation of the solution over a period of a week.

### Refinement

H-atom H2, attached to N2, was located from a difference Fourier map and refined with a distance restraint of N-H = 0.90 (1) Å. The other H-atoms were placed in idealized positions and constrained to ride on their parent atoms: C-H = 0.93 - 0.96 Å, O-H = 0.82 Å, with *U*_iso_(H) = k × *U*_eq_(parent C-, O-atom), where k = 1.5 for H-hydroxyl and H-methyl, and = 1.2 for all other H-atoms.

### Crystal data

**Table:**

C_15_H_13_BrN_2_O_3_	*D*_x_ = 1.598 Mg m^−^^3^
*M**_r_* = 349.18	Mo *K*α radiation, λ = 0.71073 Å
Orthorhombic, *P**b**c**a*	Cell parameters from 1832 reflections
*a* = 15.587 (3) Å	θ = 2.5–24.0°
*b* = 9.1281 (19) Å	µ = 2.84 mm^−^^1^
*c* = 20.399 (4) Å	*T* = 298 K
*V* = 2902.3 (10) Å^3^	Block, colourless
*Z* = 8	0.23 × 0.20 × 0.20 mm
*F*(000) = 1408	

### Data collection

**Table:**

Bruker SMART CCD area-detector diffractometer	3154 independent reflections
Radiation source: fine-focus sealed tube	1496 reflections with *I* > 2σ(*I*)
graphite	*R*_int_ = 0.074
ω scans	θ_max_ = 27.0°, θ_min_ = 2.0°
Absorption correction: multi-scan (*SADABS*; Bruker, 2001)	*h* = −19→18
*T*_min_ = 0.561, *T*_max_ = 0.600	*k* = −11→11
16311 measured reflections	*l* = −26→23

### Refinement

**Table:**

Refinement on *F*^2^	Primary atom site location: structure-invariant direct methods
Least-squares matrix: full	Secondary atom site location: difference Fourier map
*R*[*F*^2^ > 2σ(*F*^2^)] = 0.043	Hydrogen site location: inferred from neighbouring sites
*wR*(*F*^2^) = 0.127	H atoms treated by a mixture of independent and constrained refinement
*S* = 1.00	*w* = 1/[σ^2^(*F*_o_^2^) + (0.0528*P*)^2^ + 0.6195*P*] where *P* = (*F*_o_^2^ + 2*F*_c_^2^)/3
3154 reflections	(Δ/σ)_max_ = 0.001
195 parameters	Δρ_max_ = 0.64 e Å^−^^3^
1 restraint	Δρ_min_ = −0.78 e Å^−^^3^

### Special details

**Table:**

Geometry. All esds (except the esd in the dihedral angle between two l.s. planes) are estimated using the full covariance matrix. The cell esds are taken into account individually in the estimation of esds in distances, angles and torsion angles; correlations between esds in cell parameters are only used when they are defined by crystal symmetry. An approximate (isotropic) treatment of cell esds is used for estimating esds involving l.s. planes.
Refinement. Refinement of F^2^ against ALL reflections. The weighted R-factor wR and goodness of fit S are based on F^2^, conventional R-factors R are based on F, with F set to zero for negative F^2^. The threshold expression of F^2^ > 2sigma(F^2^) is used only for calculating R-factors(gt) etc. and is not relevant to the choice of reflections for refinement. R-factors based on F^2^ are statistically about twice as large as those based on F, and R- factors based on ALL data will be even larger.

### Fractional atomic coordinates and isotropic or equivalent isotropic displacement parameters (Å^2^)

**Table:**

	*x*	*y*	*z*	*U*_iso_*/*U*_eq_	
Br1	1.23601 (3)	0.49891 (6)	0.43968 (3)	0.0855 (2)	
N1	0.8301 (2)	0.4179 (3)	0.38746 (13)	0.0464 (7)	
N2	0.76415 (19)	0.4977 (3)	0.35837 (15)	0.0484 (7)	
O1	0.89179 (19)	0.2082 (3)	0.46358 (15)	0.0674 (8)	
H1	0.8543	0.2542	0.4443	0.101*	
O2	0.67329 (16)	0.3058 (3)	0.36404 (13)	0.0598 (7)	
O3	0.71532 (17)	0.6580 (3)	0.25197 (13)	0.0629 (8)	
C1	0.9761 (2)	0.4067 (4)	0.42007 (16)	0.0425 (9)	
C2	0.9676 (3)	0.2766 (4)	0.45609 (18)	0.0524 (10)	
C3	1.0386 (3)	0.2181 (4)	0.4871 (2)	0.0659 (12)	
H3	1.0329	0.1328	0.5117	0.079*	
C4	1.1175 (3)	0.2842 (5)	0.4823 (2)	0.0662 (12)	
H4	1.1644	0.2444	0.5041	0.079*	
C5	1.1271 (2)	0.4091 (5)	0.44529 (18)	0.0547 (10)	
C6	1.0566 (2)	0.4699 (4)	0.41518 (18)	0.0504 (10)	
H6	1.0632	0.5554	0.3910	0.061*	
C7	0.9037 (2)	0.4779 (4)	0.38856 (17)	0.0443 (9)	
H7	0.9113	0.5689	0.3689	0.053*	
C8	0.6876 (2)	0.4335 (4)	0.34843 (16)	0.0436 (9)	
C9	0.6191 (2)	0.5270 (4)	0.31930 (18)	0.0446 (9)	
C10	0.6332 (3)	0.6357 (4)	0.27206 (18)	0.0498 (9)	
C11	0.5635 (3)	0.7133 (4)	0.2475 (2)	0.0636 (11)	
H11	0.5723	0.7844	0.2155	0.076*	
C12	0.4824 (3)	0.6865 (5)	0.2696 (2)	0.0698 (12)	
H12	0.4368	0.7406	0.2530	0.084*	
C13	0.4671 (3)	0.5804 (5)	0.3162 (2)	0.0674 (12)	
H13	0.4117	0.5624	0.3311	0.081*	
C14	0.5358 (3)	0.5011 (4)	0.34027 (18)	0.0557 (10)	
H14	0.5259	0.4285	0.3714	0.067*	
C15	0.7306 (3)	0.7746 (5)	0.2062 (2)	0.0778 (14)	
H15A	0.7006	0.7548	0.1661	0.117*	
H15B	0.7910	0.7816	0.1975	0.117*	
H15C	0.7106	0.8653	0.2244	0.117*	
H2	0.777 (3)	0.5907 (17)	0.3488 (19)	0.080*	

### Atomic displacement parameters (Å^2^)

**Table:**

	*U*^11^	*U*^22^	*U*^33^	*U*^12^	*U*^13^	*U*^23^
Br1	0.0504 (3)	0.1095 (5)	0.0967 (4)	0.0050 (3)	−0.0067 (2)	−0.0282 (3)
N1	0.051 (2)	0.0402 (18)	0.0483 (18)	0.0045 (16)	−0.0080 (15)	0.0006 (14)
N2	0.0499 (18)	0.0346 (17)	0.0607 (19)	−0.0031 (17)	−0.0110 (15)	0.0081 (17)
O1	0.077 (2)	0.0522 (18)	0.073 (2)	0.0002 (16)	0.0031 (16)	0.0167 (15)
O2	0.0614 (17)	0.0400 (16)	0.0781 (19)	−0.0060 (13)	−0.0051 (14)	0.0116 (14)
O3	0.0557 (18)	0.0630 (18)	0.0700 (18)	0.0012 (14)	−0.0027 (14)	0.0282 (15)
C1	0.048 (2)	0.039 (2)	0.040 (2)	0.0074 (18)	−0.0037 (17)	−0.0015 (17)
C2	0.066 (3)	0.047 (3)	0.045 (2)	0.006 (2)	0.000 (2)	−0.0005 (19)
C3	0.090 (3)	0.050 (3)	0.057 (3)	0.019 (3)	−0.010 (2)	0.007 (2)
C4	0.070 (3)	0.073 (3)	0.056 (3)	0.030 (3)	−0.017 (2)	−0.015 (2)
C5	0.048 (2)	0.064 (3)	0.052 (2)	0.011 (2)	−0.0031 (19)	−0.013 (2)
C6	0.053 (3)	0.047 (2)	0.051 (2)	0.0085 (19)	−0.0009 (18)	−0.0040 (19)
C7	0.053 (2)	0.035 (2)	0.045 (2)	0.0053 (18)	−0.0015 (17)	0.0026 (17)
C8	0.052 (3)	0.036 (2)	0.043 (2)	−0.0034 (18)	−0.0039 (17)	−0.0031 (18)
C9	0.045 (2)	0.038 (2)	0.051 (2)	−0.0002 (17)	−0.0100 (17)	−0.0037 (18)
C10	0.056 (3)	0.041 (2)	0.053 (2)	0.0036 (19)	−0.010 (2)	−0.001 (2)
C11	0.064 (3)	0.057 (3)	0.070 (3)	0.006 (2)	−0.012 (2)	0.010 (2)
C12	0.060 (3)	0.063 (3)	0.086 (3)	0.015 (2)	−0.016 (2)	−0.003 (3)
C13	0.046 (3)	0.073 (3)	0.083 (3)	0.005 (2)	−0.010 (2)	−0.016 (3)
C14	0.056 (3)	0.055 (3)	0.057 (2)	−0.006 (2)	−0.0048 (18)	−0.002 (2)
C15	0.079 (3)	0.077 (3)	0.077 (3)	−0.009 (2)	−0.006 (2)	0.029 (2)

### Geometric parameters (Å, °)

**Table:**

Br1—C5	1.888 (4)	C5—C6	1.376 (5)
N1—C7	1.271 (4)	C6—H6	0.9300
N1—N2	1.393 (4)	C7—H7	0.9300
N2—C8	1.344 (4)	C8—C9	1.491 (5)
N2—H2	0.892 (10)	C9—C14	1.388 (5)
O1—C2	1.345 (5)	C9—C10	1.401 (5)
O1—H1	0.8200	C10—C11	1.390 (5)
O2—C8	1.229 (4)	C11—C12	1.364 (6)
O3—C10	1.359 (4)	C11—H11	0.9300
O3—C15	1.436 (4)	C12—C13	1.377 (6)
C1—C6	1.386 (5)	C12—H12	0.9300
C1—C2	1.403 (5)	C13—C14	1.382 (5)
C1—C7	1.451 (5)	C13—H13	0.9300
C2—C3	1.383 (5)	C14—H14	0.9300
C3—C4	1.372 (6)	C15—H15A	0.9600
C3—H3	0.9300	C15—H15B	0.9600
C4—C5	1.375 (5)	C15—H15C	0.9600
C4—H4	0.9300		
			
C7—N1—N2	116.7 (3)	O2—C8—N2	122.4 (3)
C8—N2—N1	119.4 (3)	O2—C8—C9	121.1 (3)
C8—N2—H2	125 (3)	N2—C8—C9	116.5 (3)
N1—N2—H2	115 (3)	C14—C9—C10	118.7 (3)
C2—O1—H1	109.5	C14—C9—C8	116.7 (3)
C10—O3—C15	117.6 (3)	C10—C9—C8	124.6 (3)
C6—C1—C2	118.4 (3)	O3—C10—C11	123.4 (4)
C6—C1—C7	119.1 (3)	O3—C10—C9	117.5 (3)
C2—C1—C7	122.6 (4)	C11—C10—C9	119.1 (4)
O1—C2—C3	118.2 (4)	C12—C11—C10	120.9 (4)
O1—C2—C1	122.3 (4)	C12—C11—H11	119.5
C3—C2—C1	119.5 (4)	C10—C11—H11	119.5
C4—C3—C2	120.9 (4)	C11—C12—C13	120.9 (4)
C4—C3—H3	119.5	C11—C12—H12	119.5
C2—C3—H3	119.5	C13—C12—H12	119.5
C3—C4—C5	120.1 (4)	C12—C13—C14	118.7 (4)
C3—C4—H4	119.9	C12—C13—H13	120.7
C5—C4—H4	119.9	C14—C13—H13	120.7
C4—C5—C6	119.5 (4)	C13—C14—C9	121.7 (4)
C4—C5—Br1	119.5 (3)	C13—C14—H14	119.2
C6—C5—Br1	121.0 (3)	C9—C14—H14	119.2
C5—C6—C1	121.6 (4)	O3—C15—H15A	109.5
C5—C6—H6	119.2	O3—C15—H15B	109.5
C1—C6—H6	119.2	H15A—C15—H15B	109.5
N1—C7—C1	121.1 (3)	O3—C15—H15C	109.5
N1—C7—H7	119.5	H15A—C15—H15C	109.5
C1—C7—H7	119.5	H15B—C15—H15C	109.5

### Hydrogen-bond geometry (Å, °)

**Table:**

*D*—H···*A*	*D*—H	H···*A*	*D*···*A*	*D*—H···*A*
N2—H2···O2^i^	0.89 (1)	2.14 (2)	2.978 (4)	157 (4)
N2—H2···O3	0.89 (1)	2.28 (4)	2.726 (4)	111 (3)
O1—H1···N1	0.82	1.93	2.646 (4)	146

Symmetry codes: (i) −*x*+3/2, *y*+1/2, *z*.
